# Curcumin–QingDai combination for patients with active Crohn’s disease: a retrospective, real-world multicenter cohort study

**DOI:** 10.3389/fgstr.2025.1602541

**Published:** 2025-07-15

**Authors:** Nir Salomon, Maayan Marom, Safwat Odeh, Madlen Molnar, Ariella Bar-Gil Shitrit, Omri Nayshool, Bella Ungar, Shomron Ben-Horin, Tova Rainis

**Affiliations:** ^1^ Department of Gastroenterology, Sheba Medical Center, Ramat-Gan, Israel; ^2^ Tel-Aviv University, Tel-Aviv, Israel; ^3^ Department of Gastroenterology, Bnai Zion Medical Center, Haifa, Israel; ^4^ The Rappaport Faculty of Medicine Technion-Israel Institute of Technology, Haifa, Israel; ^5^ IBD MOM Unit, Digestive Diseases Institute, Eisenberg R&D Authority, Shaare Zedek Medical Center, The Hebrew University of Jerusalem, Jerusalem, Israel

**Keywords:** Crohn’s disease, treatment, inflammatory bowel disease, complementary medicine, holistic treatment

## Abstract

**Background:**

The combination of curcumin and QingDai (CurQD) promotes aryl hydrocarbon receptor (AhR) activation and is effective in ulcerative colitis; however, its benefit in Crohn’s disease (CD) has not been studied.

**Methods:**

This is a retrospective, multicenter cohort study of patients who received CurQD for active CD, defined as the two-item patient-reported outcome (PRO2) stool frequency (SF) ≥ 2 and abdominal pain (AP) ≥ 1. The primary endpoint was the rate of clinical remission at the end of induction (weeks 8–12), defined by a PRO2 SF ≤ 1 and an AP = 0. The secondary endpoints included biomarker response [fecal calprotectin (FCP) drop ≥50% in a patient with FCP >250 μg/g at baseline] and remission (FCP drop ≥50% and FCP < 250 μg/g at the end of induction) and CurQD retention. Exploratory analysis of the public domain Genotype–Tissue Expression (GTEx) dataset was performed to elucidate the mRNA expression of AhR along the gut axis.

**Results:**

A total of 30 patients were identified for the safety dataset (13/30, 43% bio-experienced), and 25 were eligible for inclusion in the efficacy analysis. Clinical PRO2 remission at the end of induction was achieved in 12/25 (48%) patients and clinical response in 19/25 (76%), with an overall reduction in the median PRO2 score from 15 (95%CI = 13–19.7) to 4 (95%CI = 2–8.7, *p* < 0.001). Biomarker remission and response were observed in 11/20 (55%) and 15/20 (75%) patients, respectively, with baseline FCP > 250 μg/g, with an overall reduction in the median FCP from a baseline 639 μg/g (95%CI = 128–5480) to 138 μg/g (95%CI = 5–1470, *p* = 0.001). Biomarker remission was observed in 8/11 (73%) patients with colonic-involving L2/L3 disease *versus* 3/9 (33%) L1 extent (OR = 4.5, 95%CI = 0.8–24, *p* = 0.08). After 8 months’ median follow-up (range = 2–26 months), 16/19 (84%) responding patients were still taking CurQD. Three patients experienced headaches, and three had abdominal pain/diarrhea. Two of the six stopped CurQD due to these symptoms. One patient had elevated liver transaminases three times the upper limit of normal, which resolved upon halving the CurQD dose. In the public domain GTEx dataset, the mRNA expression of the target AhR in the colon *versus* the small intestinal mucosa was comparable, thereby not supporting differential AhR expression as a cause for a possible higher efficacy of CurQD in the colon,

**Conclusion:**

In this first-reported real-world experience, the AhR agonist CurQD was effective in inducing and maintaining the clinical and biomarker response and remission in over 50% of patients with CD, including in biologic-experienced patients.

## Introduction

Despite the expanding therapeutic options for the treatment of Crohn’s disease (CD), a therapeutic ceiling of efficacy is still a clinical challenge, in turn incentivizing the exploration of combination strategies to enable higher efficacy rates (1). However, most current medications are inherently immunosuppressive to a certain extent and are still costly. Thus, the long-term safety and healthcare cost considerations are barriers to combination strategies and create an unmet need for safe, affordable oral agents that can be added to the current medications.

Integrative approaches, including medical-grade nutraceuticals, are gaining increasing attention from inflammatory bowel disease (IBD) experts for their potential to complement standard medication and improve IBD care (2). A combination of two plant-based nutraceuticals, curcumin and QingDai (CurQD), has been approved as a food supplement in many countries and used as an add-on supplement in patients with ulcerative colitis (UC). In a recent modest-sized randomized placebo-controlled trial, CurQD was found superior to placebo for re-capturing clinical and endoscopic response and remission and for maintaining the response in patients with active UC, including in bio-experienced patients (3). Similar results were reported in real-world cohorts of adult and pediatric patients with active UC, including patients with moderate–severe disease and patients who failed prior advanced therapies, in whom CurQD was found effective in inducing clinical, biomarker, and/or sonographic response with overall acceptable safety profile, mostly related to transient headaches and mild liver transaminase elevation (4–7). However, in contrast to its quite extensive data in UC, there are currently no data on the efficacy of CurQD in patients with CD. Therefore, the present study aimed to describe an initial experience with CurQD in patients with active CD.

## Methods

This is a retrospective cohort chart review study of patients with CD who received treatment with CurQD at three tertiary academic centers. Charts were reviewed to extract clinical data including gender and age, disease duration, disease extent, perianal disease, history of intestinal resections, extraintestinal manifestations (EIM), and previous and concomitant medications. To be included, patients had to have CD of at least 3 months’ duration diagnosed based on conventional clinical, endoscopic, and histologic criteria. All patients fulfilling this CD diagnostic criteria who received CurQD were included in the safety analysis. To be eligible for the efficacy analysis, patients had to start CurQD after being on stable medical treatment for at least 8 weeks and to have had an active disease at baseline, defined by a two-item patient-reported outcome (PRO2) stool frequency (SF) of ≥2 and abdominal pain (AP) ≥1 (8, 9) The primary endpoint was the rate of clinical remission at the end of induction by weeks 8–12, defined as PRO2 SF of ≤1 and AP = 0 (8, 10). The secondary endpoints included clinical response defined as a PRO2 weighted score drop of ≥5 points, corresponding to a CDAI 70 change (8), biomarker response [fecal calprotectin (FCP) drop ≥50% in a patient with FCP > 250 μg/g at baseline], and remission (FCP drop ≥50% and FCP < 250 μg/g), as well as safety of CurQD. Drug retention at the end of follow-up was another predefined secondary endpoint.

CurQD was purchased from Evinature Ltd. (Binyamina, Israel) after manufacturing at a Good Manufacturing Practice (GMP) facility. All batches were tested for active moiety content by LC-MS/MS for indigo and indirubin concentrations corresponding to their levels in batches used in a previous placebo-controlled clinical trial (4).

### GTEx data analysis

To explore the mRNA expression of aryl hydrocarbon receptor (AhR) in the colon, small bowel, and whole blood, data from the Genotype–Tissue Expression (GTEx) database were used. The GTEx Portal (v8, dbGaP accession phs000424.v8.p2), accessed on May 6, 2024, was queried for normalized expression levels of AhR mRNA across relevant tissue types. Specifically, expression data for the colonic mucosa, small bowel mucosa, and whole blood were extracted, and differential expression analysis was performed to compare variations in the mRNA levels of AhR within these tissues. The expression levels were visualized using bulk RNA sequencing data provided by the GTEx database. Statistical significance was determined using analysis of variance (ANOVA) followed by a *post-hoc* test for pairwise comparisons.

### Statistical analysis

Descriptive statistics were performed using medians and 25%–75% interquartile ranges (IQRs) for continuous variables and percentages for categorical variables. The Wilcoxon paired test was used to compare pre–post treatment continuous variables, while Fisher’s exact test with calculation of odds ratios (ORs) and 95% confidence intervals (95%CIs) was used to compare categorical parameters. Backwards regression analysis was employed to perform a multivariable analysis of the variables associated with the primary outcome with a *p*-value <0.1 and with disease duration. A non-responder imputation was applied, whereby patients with missing outcome data, patients lost to follow-up, or patients who stopped treatment due to any adverse event were considered as having had a non-response to CurQD. The study was approved by the institutional review boards of the participating centers.

## Results

Between July 2022 and September 2024, 30 patients with CD who received add-on CurQD were identified. The background characteristics of these patients are shown in **Table 1**. Disease duration was ≥2 years in 22/30 (73%) patients, and 13/30 (43%) patients were bio-experienced. In 9/30 (30%) patients, CurQD was added as a combination with an ongoing concurrent biologic due to active disease despite stable dose biologic treatment (three with adalimumab, two each with infliximab and vedolizumab, and one each with ustekinumab and certolizumab), while it was administered in four patients while they were not responding to ongoing corticosteroids.

**Table 1:** Baseline characteristics of the study cohort (full safety dataset, *n* = 30).

Median age (years) (25%–75% IQR)	33 (23–47)
Women	16 (53%)
Median disease duration (years) (25%–75% IQR)	4 (1.2–8)
Extraintestinal manifestations	12 (40%)
Disease extent	
Small bowel (L1)	16 (53%)
Colonic (L2)	7 (23%)
Ileo-colonic (L3)	7 (23%)
Perianal disease	8 (26.6%)
Previous intestinal surgery	1 (3%)
Bio-experienced^a^	13 (43%)
Concomitant biologics/small molecule	9 (30%)
Concomitant corticosteroids	4 (13%)

a Bio-experienced includes patients with previous exposure to biologics and/or small molecules.

The induction dosing of CurQD comprised a QD-predominant formulation (>50% of capsule content) and ranged between 1 and 2 g per day in two divided doses. The dosing was determined by the treating integrative medicine practitioners (NS or MM) based on the severity of disease activity and weight. In patients achieving clinical remission, the formulation was gradually reduced toward the lowest QD/curcumin ratio that maintained the clinical response. Of the 30 patients comprising the exposed safety set, four patients were excluded from the efficacy analysis due to starting CurQD in the absence of predefined active disease. One additional patient started CurQD due to active CD symptoms 4 weeks after starting an adalimumab biosimilar and 1 week after stopping corticosteroids. He had a good response and was maintained on adalimumab+CurQD until the end of follow-up at 7 months. However, because this did not comply with the predefined requirement for stable background medications, this patient was also excluded from the efficacy dataset. Thus, 25 patients remained for the efficacy analysis, of whom 20 (80%) had baseline FCP > 250 μg/g. In 11/25 (44%) patients, a baseline endoscopy (lower endoscopy and/or capsule endoscopy) was available within 2 months before starting CurQD and showed active inflammation in all but one patient in whom capsule endoscopy only showed mild hyperemia in the distal ileum in the presence of FCP of 128 μg/g.

### Efficacy results: induction

**Figure 1:**
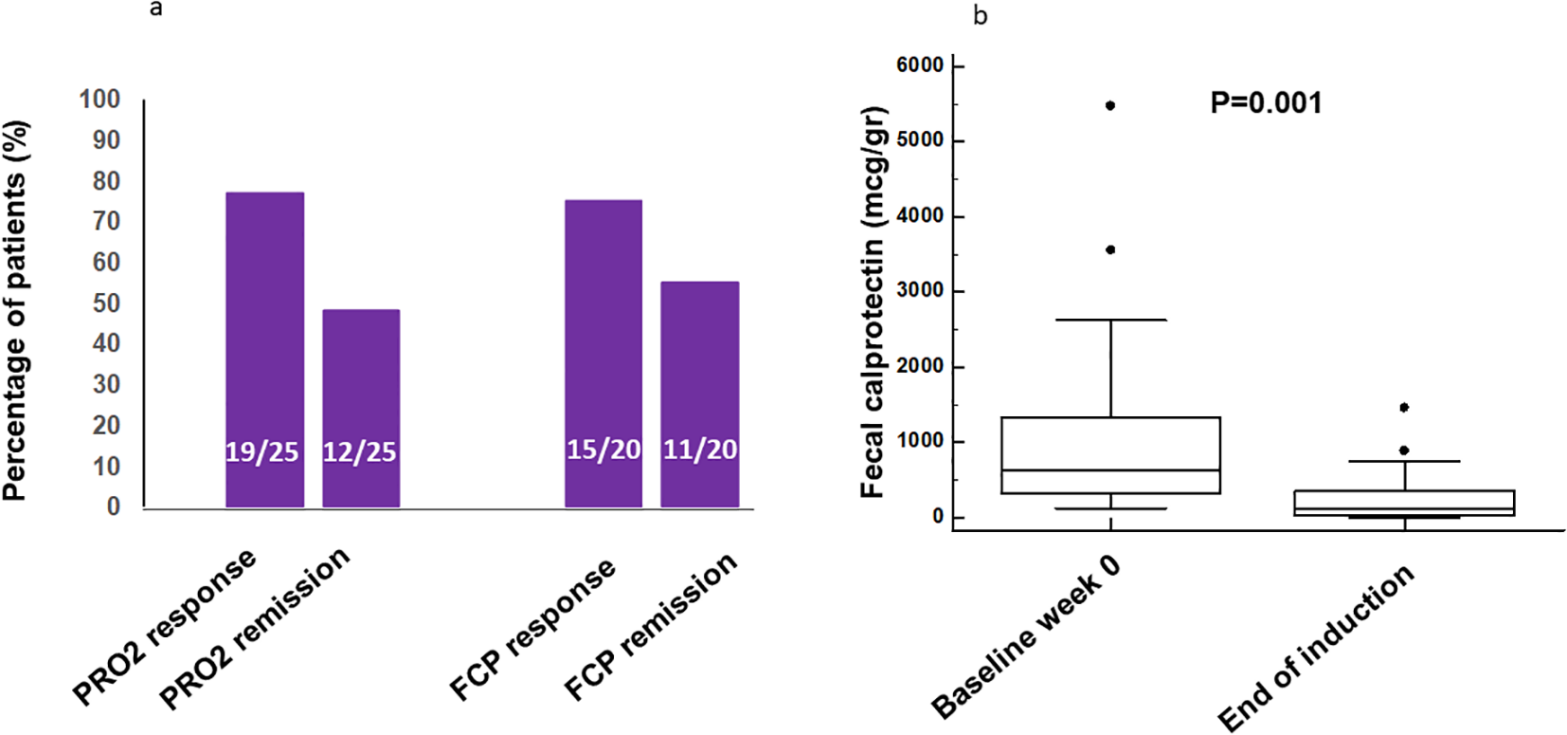
**(a)** Rates of end-of-induction clinical two-item patient-reported outcome (PRO2) remission and response in the efficacy dataset (*n* = 25) and of calprotectin remission and response in the efficacy dataset in patients with baseline calprotectin >250 μg/g (*n* = 20). **(b)** Median calprotectin levels in stool at baseline and at the end of induction. *FCP*, fecal calprotectin.

**Figure 2:**
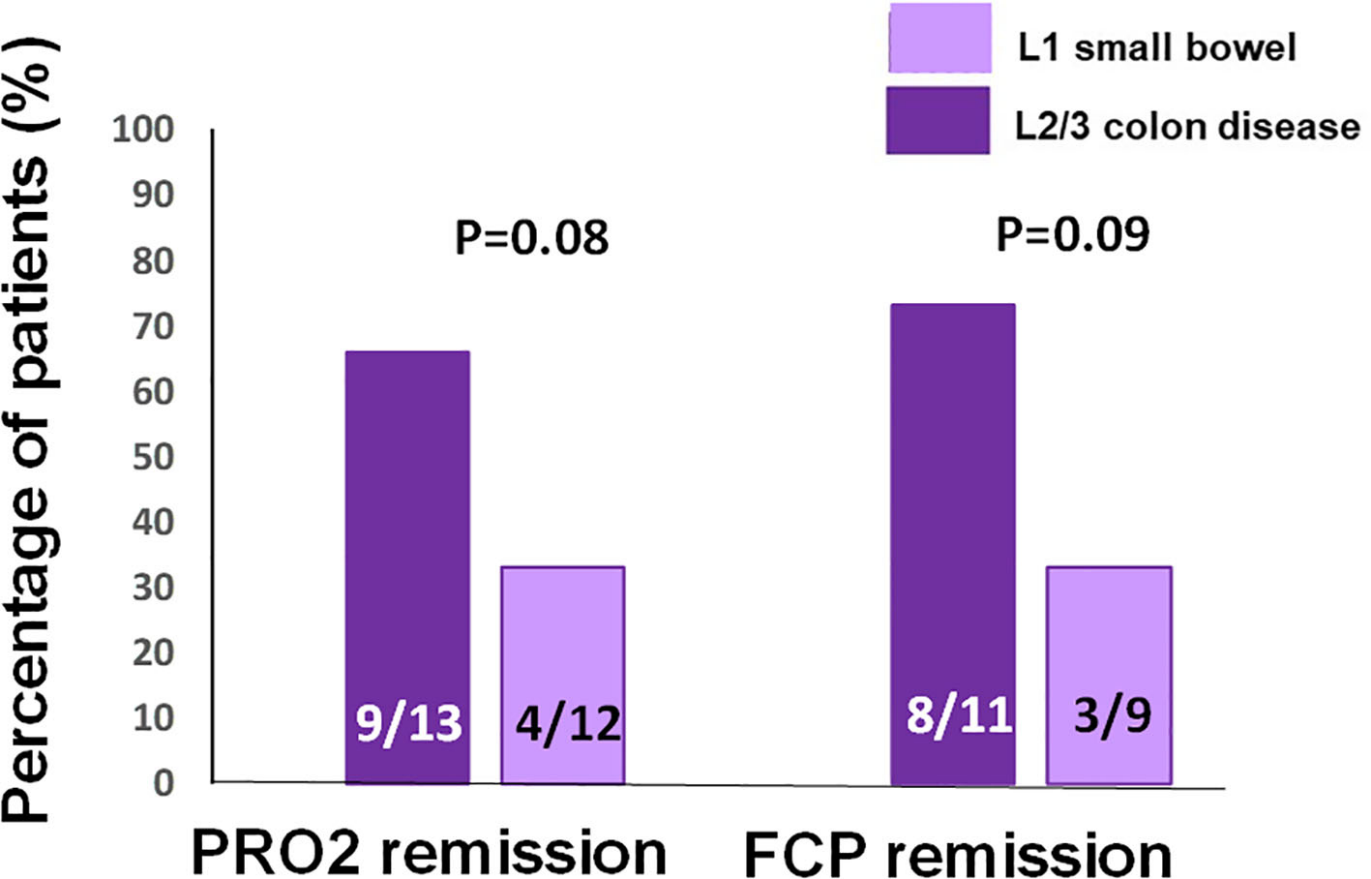
Rates of the two-item patient-reported outcome (PRO2) remission and biomarker calprotectin remission at the end of induction in patients with L1 small bowel Crohn’s disease (CD) *versus* those with disease involving the colon (L2 or L3).

At the end of induction, clinical remission (PRO2 SF of ≤1 and AP = 0) was achieved in 12/25 (48%) patients and PRO2 clinical response in 19/25 (76%) patients, with an overall reduction in the median PRO2 score from 15 (95%CI = 13–19.7) to 4 (95%CI = 2–8.7, *p* < 0.001) (**Figure 1**, **Supplementary Figure S1**). Four of the six PRO2 non-responders had a low FCP of <250 μg/g at baseline. Calprotectin was reduced from a median baseline value of 639 μg/g (95%CI = 128–5,480) to 138 μg/g (95%CI = 5–1,470, *p* = 0.001) (**Figure 2**). Among the 20 patients with a baseline FCP > 250 μg/g, biomarker remission and response were observed in 11 (55%) and 15 (75%), respectively. Four patients were on concomitant corticosteroids when starting CurQD, and three of these patients were weaned successfully and achieved steroid-free remission at the end of induction.

On a subgroup analysis according to disease extent, 9/13 (66%) patients with ileocolonic or colonic disease achieved clinical remission compared with 4/12 (33%) patients with small bowel L1 disease (OR = 4.5, 95%CI = 0.8–24, *p* = 0.08) (**Figure 2**). Biomarker remission was observed in 8/11 (73%) patients with colonic-involving L2 or L3 disease compared with 3/9 (33%) patients with L1 disease (OR = 5.3, 95%CI = 0.8–36, *p* = 0.09).

On a subgroup analysis, 5/11 bio-experienced *versus* 8/14 bio-naive patients achieved clinical remission (OR = 0.6, 95%CI = 0.1–3.1, *p* = 0.6). Among the patients with a baseline FCP >250 μg/g, 7/9 (78%) bio-experienced patients achieved biomarker remission *versus* 4/11 (36%) bio-naive patients (OR = 2.1, 95%CI = 0.9–5, *p* = 0.07). In a logistic regression analysis, L2/L3 disease extent and being bio-experienced retained a borderline statistical independent association with biomarker remission (**Table 2**).

**Table 2:** Multivariable analysis of the factors associated with biomarker calprotectin remission among the efficacy dataset patients who had baseline calprotectin >250 μg/g (*n* = 20).

	Odds ratio	95% Confidence interval	*p*-value
Colonic-involving disease	40	0.8–2038	0.07
Being bio-experienced	90	0.85–9520	0.06
Disease duration	0.8	0.6–1.1	0.16

### Efficacy results: maintenance

**Figure 3:**
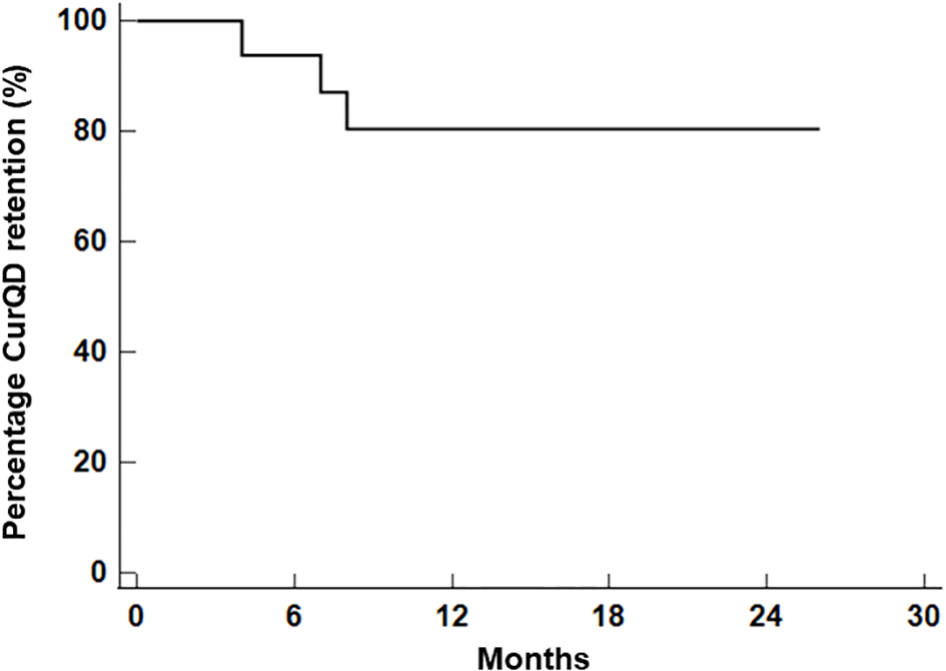
Kaplan–Meier curves of curcumin and QingDai (CurQD) drug retention among patients responding to CurQD at the end of induction.

Of 25 patients, 19 (64%) continued CurQD as maintenance combination therapy with their background medications, including biologics. Four patients stopped CurQD during induction due to inefficacy, while two stopped due to adverse events. After a median follow-up of 8 months (25%–75% IQR = 4.5–10, range = 2–26 months), 16/19 (84%) patients were still taking CurQD at a tapered maintenance dose (**Figure 3**). Nine patients flared while on maintenance dose, and all responded to the re-induction dose.

### Safety analysis

Of the 30 patients in the safety dataset, 7 (23%) experienced adverse events. Three patients experienced headaches and three had abdominal pain or diarrhea. Two of these six patients stopped CurQD due to these symptoms, although the severity of symptoms was judged to be mild to moderate in all cases. One patient had elevated liver transaminases three times the upper limit of normal (ULN), which resolved upon halving the CurQD dose.

### mRNA expression of AhR in the colon and small bowel

**Figure 4:**
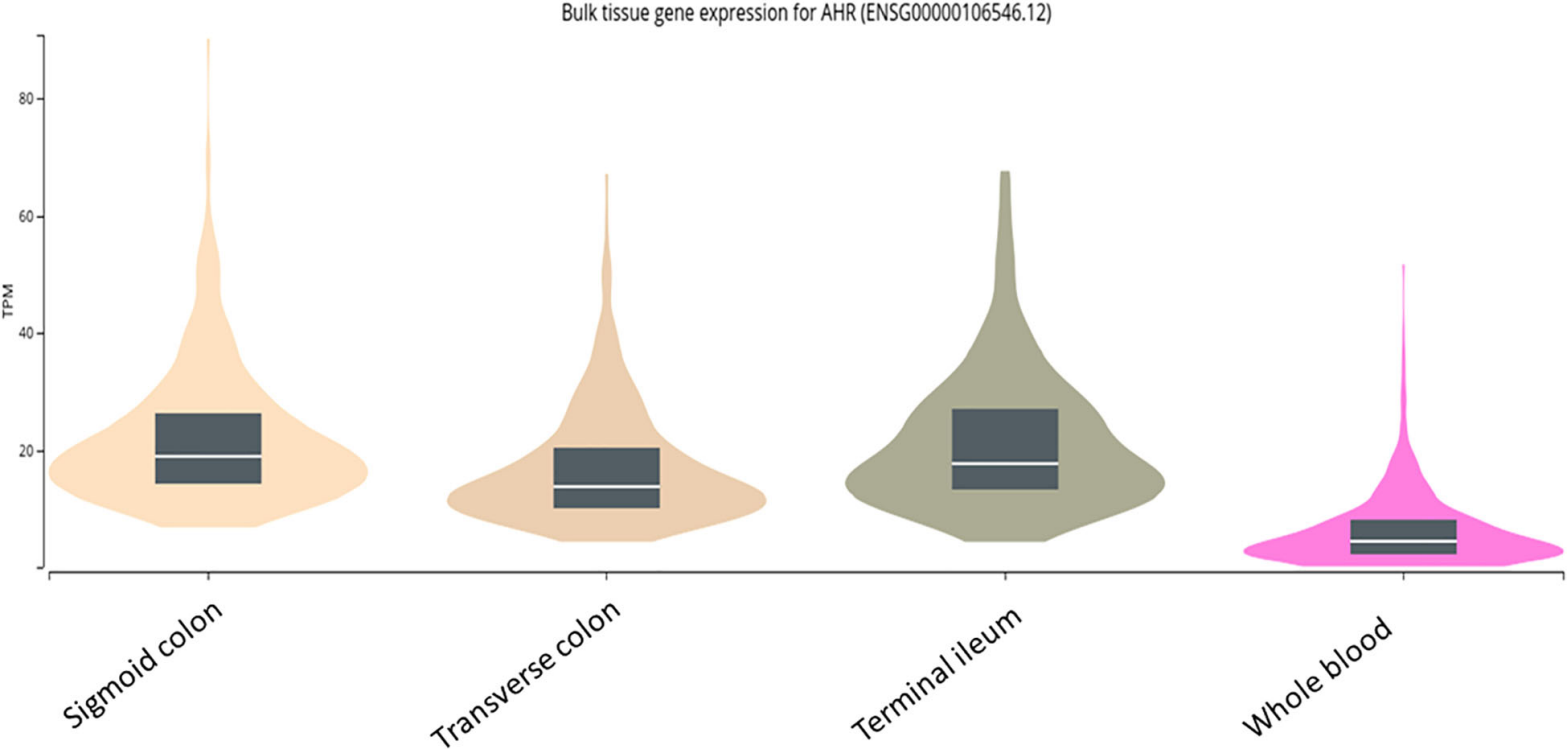
mRNA expression of aryl hydrocarbon receptor (AhR) in the human mucosa of the terminal ileum *versus* two colonic segments and *versus* whole blood as extracted from the public domain Genotype–Tissue Expression (GTEx) dataset.

Due to the observed trend for better biomarker and clinical response among patients with L2/L3 disease, and because CurQD is known to activate the AhR pathway (4), we were interested to explore whether this signal may stem from a higher AhR density in the human colon compared with its expression level in the small bowel. Therefore, the public domain GTEx dataset was assessed for AhR expression in healthy colonic *versus* small bowel mucosa and *versus* whole blood. The results showed that the mRNA expression of AhR in humans was comparable between the colon and the small bowel, and both were significantly elevated compared with the whole blood bulk RNA expression of this receptor (**Figure 4**).

## Discussion

Despite the advent of biologics and small molecule agents for CD, novel combination strategies to improve patient outcomes are still being investigated. This study explored the addition of CurQD, a nutraceutical formulation derived from traditional Chinese Medicine, in patients with CD who had inadequate response or who had loss-of-response to their current medications, which showed its efficacy in inducing clinical and biomarker remission in over half of the patients.

There is increasingly compelling data on the efficacy of CurQD in both adult and pediatric patients with active ulcerative colitis, and it has been adopted for this indication by IBD experts in the USA, Israel, and elsewhere (3–7, 9, 11, 12). However, to date, no data on its potential benefit in CD have been published. The present report is the first to suggest a potential efficacy in active CD, whereby over half of the patients attained clinical remission with CurQD added on to a CD treatment. Interestingly, similar to previous observations in UC [3.4], there was no difference in the response to CurQD between bio-naive and bio-experienced patients in the present study (**Table 2**), possibly owing to its AhR agonistic mechanism of action that is believed to be more involved in mucosal epithelial reconstitution and immunoregulation rather than in immunosuppression per se (13). However, larger studies are needed to ascertain whether the effect of CurQD in CD is independent of the prior failure of biologics and small molecules.

One of the observations in the present study was a signal for a better biomarker remission for patients with CD involving the colon compared with isolated small bowel disease. It is known that the genetic–transcriptomic profile of colonic CD resembles more closely UC than small bowel CD (14). Thus, we were interested to see whether this signal stems from differential expression patterns along the intestine of CurQD target, namely, the AhR molecule, which is activated in the mucosa of patients treated with CurQD (4). However, investigation of the public domain dataset in the GTEx repository indicated that the AhR receptor is similarly expressed in the small and large intestines of healthy individuals. Two previous studies implicated AhR in the pathogenesis of CD by showing a decreased AhR signaling in tissue-residing ILC-3 cells in the terminal ileum of patients with CD, leading to increased ILC3-to-ILC1 phenotypic conversion and the exacerbation of intestinal inflammation (15, 16). Collectively, these mechanistic-level data suggest that CD inflammation of the small bowel may be just as amenable to the beneficial effect of AhR activation exerted by CurQD as its colonic counterpart. Therefore, it may be that the signal for a better efficacy in colonic CD in the present study merely reflects the more resistant nature and lower response rate to any therapeutics of small bowel CD compared with colonic CD, as was found across different biologics (17). Moreover, the signal for a better efficacy of CurQD in colonic-involving disease did not reach statistical significance, and the clinical remission rates were comparable. Therefore, future studies are needed to corroborate whether a differential locoregional effect is indeed present.

Two of the patients had to stop CurQD due to nonspecific abdominal and headache complaints. One patient had mild elevation of liver transaminases, which resolved once the dose was halved. Therefore, monitoring liver tests during the first 8 weeks of CurQD induction is recommended. QingDai has on occasion been associated with allergic ischemic colitis and intussusception, but this was not observed in our patients. The safety of QingDai in Southeast Asian populations has drawn attention due to potential rare pulmonary hypertension (PHT) in patients taking prolonged large doses (18). In the present study, which included patients taking CurQD for over a year and a few over 2 years, no PHT was reported, and neither was this detected in multiple previous cohorts receiving this nutraceutical (3–5, 7). This may be due to the deliberate CurQD protocol used, whereby the ratio of QD/curcumin was reduced as the patients’ disease activity improved (down to the lowest QD/curcumin ratio able to maintain remission). Conversely, the lack of observed PHT with CurQD may be due to different sourcing of the compounds and/or the stringent GMP standards of manufacturing. However, continued surveillance is still warranted.

Several limitations of the present study should be noted, including its retrospective uncontrolled nature, the small cohort size, the absence of well-documented data regarding the EIM response, and the lack of objective endoscopic or sonographic data to support the biomarker data.

In conclusion, in this first report, the addition of CurQD as a combination with current medications was found to be effective in inducing and maintaining clinical and biomarker remission in patients with active CD, including in patients failing biologics and/or small molecules.

## Data Availability

The raw data supporting the conclusions of this article will be made available by the authors, without undue reservation.

## Supplementary material

Supplementary Figure 1 Median PRO2 scores at baseline (starting CurQD as add-on combination to current stable medications), and at the end of induction at week 8–12.
