# Supplementary figures and images for: Curcumin–QingDai combination for patients with active Crohn’s disease: a retrospective, real-world multicenter cohort study
Source: Front Gastroenterol (Lausanne). 2025 Jul 15;4:1602541. doi: 10.3389/fgstr.2025.1602541 (PMC12952403; doi:10.3389/fgstr.2025.1602541)

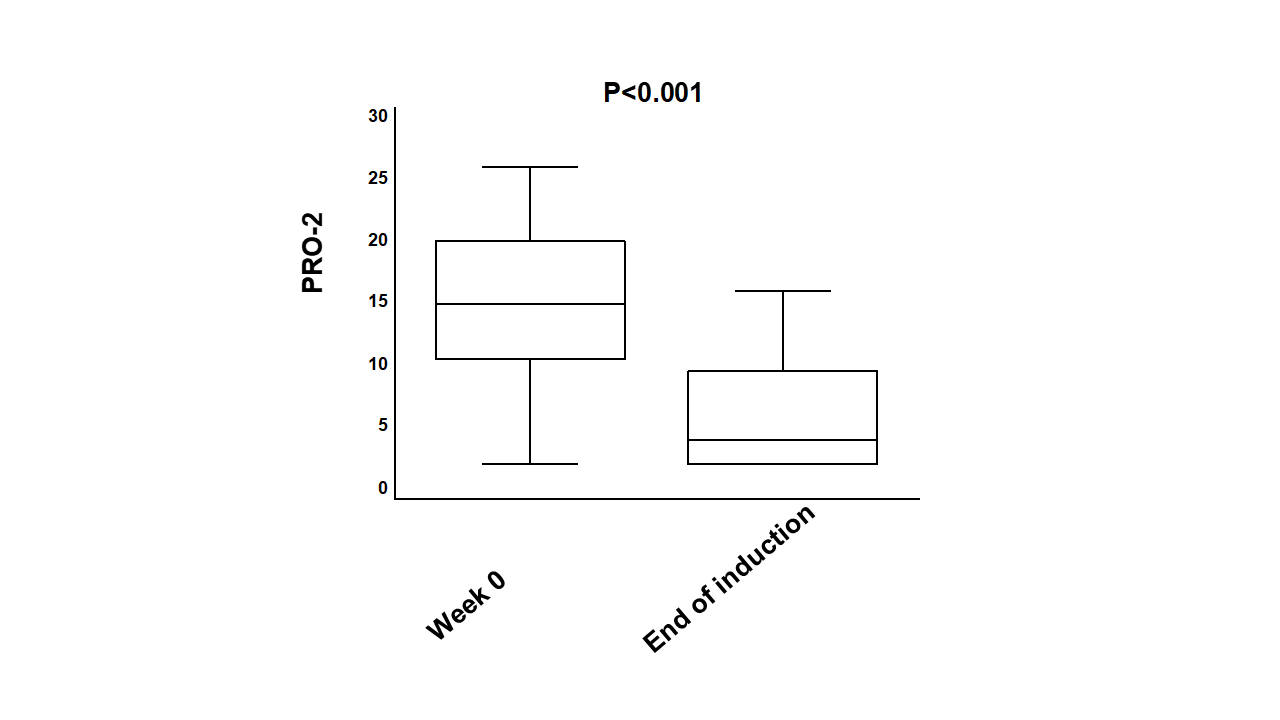

Supplement: Supplementary Figure 1 — Median PRO2 scores at baseline (starting CurQD as add-on combination to current stable medications), and at the end of induction at week 8–12. [file Image1.tif]
